# Entering a new era of antiplatelet therapy: the 4D-ACS trial

**DOI:** 10.1093/ehjcvp/pvaf045

**Published:** 2025-06-03

**Authors:** Claudio Laudani, Felice Gragnano, Mattia Galli

**Affiliations:** Division of Cardiology, Azienda Ospedaliero-Universitaria Policlinico ‘Rodolico—San Marco’, University of Catania, Catania 95100, Italy; Division of Cardiology, University of Florida College of Medicine, Jacksonville, FL 32209, USA; Department of Translational Medical Sciences, University of Campania “Luigi Vanvitelli”, Caserta 81100, Italy; Division of Cardiology, A.O.R.N. “Sant'Anna e San Sebastiano”, Caserta 81100, Italy; Department of Medical-Surgical Sciences and Biotechnologies, Sapienza University of Rome, Corso della Repubblica, 79, Latina 04100, Italy; Cardiovascular Department, Maria Cecilia Hospital, GVM Care & Research, Cotignola 48033, Italy

De-escalation of dual antiplatelet therapy (DAPT) after acute coronary syndrome (ACS) or percutaneous coronary intervention (PCI) can be achieved either by discontinuing one antiplatelet agent (aspirin or the P2Y_12_ inhibitor) or by mitigating P2Y_12_ inhibiting therapy through dose reduction (e.g. lowering the dose of prasugrel or ticagrelor) or switch (from prasugrel or ticagrelor to clopidogrel).^1^ The clinical impact of each de-escalation strategy varies depending on the specific approach used, the clinical setting (acute vs. chronic), the type of P2Y_12_ inhibitor, and patient’s characteristics such as ethnicity or sex.^1–4^

The 4D-ACS trial explored whether a ‘dual de-escalation strategy’ combining aspirin withdrawal and prasugrel dose reduction (from 10 to 5 mg daily) could offer additional benefit over prasugrel dose reduction alone 1 month post-ACS.^5^ A total of 656 ACS patients undergoing drug-coated stent implantation were randomized to either 1-month DAPT with aspirin 100 mg and prasugrel 10 mg (or 5 mg in patients aged ≥75 years or body weight <60 kg) followed by prasugrel 5 mg monotherapy (1M-DAPT group) or 12-month DAPT with aspirin and prasugrel 5 mg (12M-DAPT group).^5^ At 12 months, the 1M-DAPT group strategy was both non-inferior and superior to the 12M-DAPT group strategy, resulting in a 49% relative risk reduction in net adverse clinical events (4.9% vs. 8.8%), defined as the composite of death, myocardial infarction, stroke, ischaemia-driven target vessel revascularization, and Bleeding Academic Research Consortium (BARC) 2–5 bleeding. This benefit was primary driven by an 87% relative reduction in BARC 3 or 5 bleeding events (0.6% vs. 4.6%). Ischaemic outcomes were similar in the two groups (2.4% vs. 3.7%).^5^ Limitations of the study include its small sample size, non-inferiority design, and the predominantly male (80%) East-Asian population, which may limit generalizability. Moreover, the trial lacks a standard therapy arm. The ongoing DESC-HBR (NCT05903976) and ELECTRA-SIRIO-2 (NCT04718025) trials are expected to provide further insights into the dual de-escalation strategy, combining aspirin withdrawal with dose reduction of ticagrelor or prasugrel following a short course of standard DAPT. Meanwhile, the NEOMINDSET (NCT04360720) and LEGACY (NCT05125276) trials will evaluate the safety and efficacy of aspirin withdrawal at the time of PCI while maintaining full-dose ticagrelor or prasugrel, compared with standard DAPT.

## Data Availability

No new data were generated or analysed in support of this research.
